# Transcription factor E2F3a regulates *CASP8AP2* transcription and enhances sensitivity to chemotherapeutic drugs in acute lymphoblastic leukemia

**DOI:** 10.1186/s12935-018-0531-1

**Published:** 2018-03-20

**Authors:** Fei-Fei Liu, Kai-Ling Wang, Li-Ping Deng, Xiao Liu, Min-yuan Wu, Tian-You Wang, Lei Cui, Zhi-Gang Li

**Affiliations:** 1 Hematology & Oncology Laboratory, Beijing Pediatric Research Institute, Beijing Children’s Hospital, Capital Medical University, National Center for Children’s Health; Beijing Key Laboratory of Pediatric Hematology Oncology; Key Laboratory of Major Diseases in Children, Ministry of Education; National Key Discipline of Pediatrics, Ministry of Education, Beijing, China; 2 Hematology & Oncology Center, Beijing Children’s Hospital, Capital Medical University, National Center for Children’s Health; Beijing Key Laboratory of Pediatric Hematology Oncology; Key Laboratory of Major Diseases in Children, Ministry of Education; National Key Discipline of Pediatrics, Ministry of Education, Beijing, China; 3 Present Address: Department of Pediatrics, Affiliated Hospital of Binzhou Medical University, Binzhou, 256603 Shandong Province China; 4 Present Address: Department of Pediatrics, Beijing Luhe Hospital, Capital Medical University, 82 Xinhua Nan Road, Tongzhou District, Beijing, 101149 China

**Keywords:** Childhood acute lymphoblastic leukemia, E2F3a, CASP8AP2, Transcription regulation, Chemotherapeutic sensitivity

## Abstract

**Background:**

Low expression of *E2F3a* and caspase 8 associated protein 2 (*CASP8AP2*) are associated with poor prognosis of childhood acute lymphoblastic leukemia (ALL).

**Methods:**

Dual-luciferase reporter assay and wild type as well as four mutated types of reporter plasmids were used to demonstrate the activation of E2F3a on *CASP8AP2* transcription. The direct binding of E2F3a with the promoter of *CASP8AP2* was shown by Chromatin Immunoprecipitation (ChIP). Cell proliferation activity and cell cycle were determined by MTS and flow cytometry in leukemic cells after treating with common chemotherapeutic drugs vincristine and daunorubicin.

**Results:**

In this study, we found that up-regulation of E2F3a in leukemic cells led to increased fraction of cells in S and G2/M phase, accelerated proliferation, and enhanced sensitivity to vincristine and daunorubicin. ChIP and luciferase assay indicated that E2F3a could directly bind to two fragments in the wild type of *CASP8AP2* promotor (− 206 to − 69 and − 677 to − 507), and activate its transcription activity which was reduced in mutated promotors. The effect of E2F3a on chemotherapeutic sensitivity of leukemic cells could be reversed by down-regulating *CASP8AP2*.

**Conclusions:**

E2F3a could promote transcription and expression of *CASP8AP2*. The effect of E2F3a on chemotherapeutic sensitivity of ALL cells was implemented by regulating *CASP8AP2* expression to a great extent.

## Background

Leukemia is the most common malignancy in children [1]. Hereinto, acute lymphoblastic leukemia (ALL) is about 70%. In recent 30 years, 5-year event free survival (EFS) has been more than 80% based on risk-adapted chemotherapy. However, 10% of the children with ALL relapse eventually [2]. Relapse has been the main obstacle to further improvement of prognosis in childhood ALL [3].

Caspase 8 associated protein 2 (CASP8AP2) is a multifunctional protein participating in apoptosis mediated by FAS and tumor necrosis factor α [4], S phase progression [5], transcription regulation mediated by corticoid receptor and c-Myb [6], transcription and 3′ end processing of replication dependent histone mRNA [7]. In childhood ALL, it was found that low expression of *CASP8AP2* mRNA was correlated to high level of minimal residual disease (MRD) and relapse [8–11]. However, it was unclear about the mechanisms of down-regulation of *CASP8AP2* in childhood ALL.

E2F family was thought of as one of the main transcription regulators of replication dependent histone, and played an important role in regulation of cell cycle progression [12]. Furthermore, the members of E2F family have been reported to be involved in genesis and progression of many types of tumors [13, 14]. Gene expression profiling in childhood ALL showed *E2F3* expressed in most patients, whereas other members’ expression was low or undetectable [15, 16]. There were two E2F3 isoforms, E2F3a and E2F3b [17, 18]. The latter could be found in G0 phase, whereas E2F3a played a pivotal role in transition of G1/S and cell proliferation [19]. Our previous study has showed that similar to *CASP8AP2*, low expression of *E2F3a* was common and related to poor treatment outcome in childhood ALL [20].

In the present study, we showed that E2F3a could activate *CASP8AP2* transcription directly; its over-expression enhanced the sensitivity of leukemic cells to chemotherapeutic drugs, and could be counteracted by knock down of *CASP8AP2*. Thus, in childhood ALL, the prognostic significance of *E2F3a* was largely implemented through regulating *CASP8AP2* expression.

## Methods

### Plasmids

Recombinant plasmid pcDNA3-E2F3a was a kind gift from Professor Kiyoshi Ohtani at Kansai University, Japan. shCASP8AP2, a tetracycline-inducible short hairpin RNA (Tet-on shRNA) expression plasmid, was kindly given by Professor Vincenzo de Laurenzi of Sapienza University of Rome, which was generated from human *CASP8AP2* nucleotides 416 to 434 (5′-GATTGTCTGAGTTTCCACA-3′). Tet-on recombinant lentivirus for over-expression of E2F3a and negative control (lentivirus encoding red fluorescent protein) were products of Shanghai Genechem Co., Ltd.

There were three E2F binding sites (TTTSSCGC) in the 1000 bp sequence upstream of the transcription start site of *CASP8AP2* (GenBank AL353692.14), at − 614, − 169, − 131 respectively. The DNA fragment containing these three binding sites was amplified using 5′-CATATGCCATGGGCCCCTATGACAACTTACCC-3′ as forward primer and 5′-CGAATAAGATCTGGTGGCCTTATTTTCGCACC-3′ as reverse primer. Underlined letters represented the two introduced restriction digestion sites (*Nco*I in forward primer and *Bgl*II in reverse primer respectively). After digested by *Nco*I and *Bgl*II, PCR product was subcloned into vector pGL3-basic to make luciferase reporter CASP8AP2-wild.

**Fig. 1:**
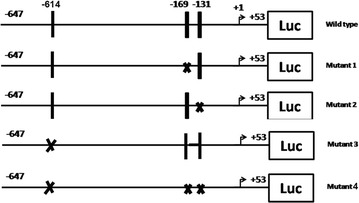
Diagram of the wild type and 4 types of mutant promoters of *CASP8AP2*. The vertical lines represented the binding sites of E2F3a. The crosses represented the mutated binding sites. The arrow represented the transcription start site. *Luc* luciferase

Based on CASP8AP2-wild, four mutated reporters were generated using Fast Mutagenesis System (TransGen Biotech Co., Ltd, China): CASP8AP2-mutant1, -mutant2, -mutant3, -mutant4, which were mutated at binding site − 169, − 131, − 617, and all the three sites respectively (Fig. 1). All binding sites in the four reporters were mutated to 5′-ctctctct-3′ confirmed by sequencing. The primers used in mutagenesis were listed in Table 1.

**Table 1:** The Primers used in mutagenesis

Mutation sites	Primers
− 169	F: 5′-AGGAAGATTACTGGAGCTCTCTCTCAACCGTGTTTTTTTTTT-3′
	R: 5′-AGAGAGAGCTCCAGTAATCTTCCTAGGGCTTTAAGAG-3′
− 131	F: 5′-TTTTTTCCCTTCCAAAGCTCTCTCTTAGTTGTGGGCGCTG-3′
	R: 5′-AGAGAGAGCTTTGGAAGGGAAAAAAAAAAACACGGTTG-3′
− 614	F: 5′-AGATCTGGTGGCCTTATCTCTCTCTCTATCAATGCTCGGG-3′
	R: 5′-AGAGAGAGATAAGGCCACCAGATCTCGAGCCCGGGCTA-3′

### Cell lines

Human ALL cell lines 697, REH, MHH-CALL-2, Nalm-6 cells were cultured in RPMI 1640 (GIBCO, USA) supplemented with 10% fetal bovine serum (FBS) and penicillin/streptomycin. Human leukemia SUP-B15 cells were cultured in Iscove’s modified Dulbecco’s media (IMDM) supplemented with 20% FBS. Human colorectal cancer HCT116 cells were cultured in DMEM supplemented with 10% FBS. All cells were maintained at 37 °C in a humidified atmosphere containing 5% CO_2_.

### Transfection

To transfect plasmids, Lipofectamine 2000 (Invitrogen, USA) and ECM830 Electroporation System (BTX, USA) were used for HCT116 and human ALL cell lines respectively.

### Dual-luciferase reporter assay

Transfected cells were collected after 48 h. Activities of firefly luciferase (FL) and renilla luciferase (RL) were determined using Dual-Luciferase^®^ Reporter Assay System (Promega, USA). Relative luciferase activity was represented by FL/RL.

### Determination of cell proliferation

Cell proliferation was determined by MTS method. Briefly, cells were plated into 96-well plate, 100 μl/well. For HCT116, cell confluence should be about 30%; for leukemic cells, the number of cells should be about 10^4^ per well. After treatment with Doxycycline (0.5 μg/ml), chemotherapeutic drugs (10^−4^, 10^−3^, 10^−2^, 10^−1^, 1, 10 μg/ml of Vincristine or Daunorubicin), or plasmid transfection, the cells were cultured for 24 h at 37 °C in a humidified atmosphere containing 5% CO_2_. Twenty microlitre of CellTiter 96 AQueous One Solution Reagent (Promega, USA) was added to each well and mixed gently. After incubation for 2–4 h, absorbance at 490 nm was determined using microplate reader. After calibrated with non cellular background, cell viability was calculated using a non treatment control regarded as 100% of cell viability.

### E2F3a stable infection

697 and REH cells were infected by inducible Tet-on E2F3a lentivirus (multiplicity of infection = 100). Puromycin (0.4 μg/ml) was added after 48 h. Culture medium was changed every two days until the sixth or eighth day, at that time the stable infected cells were screened out. These cells could be induced to over-express *E2F3a* by Doxycycline (Dox, 0.5 μg/ml).

### Determination of cell cycle phases

Flow cytometry was used to determine the changes in cell cycle phase fractions. Briefly, Dox was firstly tested for its effect on cell cycle phrase fractions in 697 cell. After serum deprivation and induction of *E2F3a* over-expression by Dox, the 697 cell stably infected with inducible Tet-on E2F3a lentivirus was cultured for 24 h in complete medium containing 10% fetal bovine serum. The harvested cells were washed using phosphate-buffered saline, fixed by 75% ethanol, and digested by RNase A. After nucleus staining by 5 μl of propidium iodide (PI), the cell cycle phase fractions was determined by flow cytometry.

### Chromatin Immunoprecipitation (ChIP)

The ChIP assay was carried out with ChIP assay kit (Beyotime Institute of Biotechnology, China), anti-E2F3 or rabbit IgG antibody. The separated DNA was amplified with the PCR primer pairs, 5′-GCCCCTCTCTTAAAGCCC-3′ and 5′-GAGTGCCTACGGCCAATCAT-3′ for fragment − 206 to − 69, 5′-GGGTGGAATACCTGACTTGC-3′ and 5′-GCAAGTCAGGTATTCCACC-3′ for fragment − 677 to − 507 in *CASP8AP2* promotor respectively.

### Western blotting

Total proteins were extracted from cells lysed with RIPA lysis buffer and separated using SDS-PAGE. Nuclear proteins were separated with NE-PER^®^ Nuclear and Cytoplasmic Extraction Reagents (Thermo Fisher Scientific, USA) and NuPAGE Bis–Tris Mini Gels System (GIBCO, USA). Western blotting was carried out with antibodies described below.

### Antibodies

The antibodies used in this study included anti-E2F3 (Abcam, UK), rabbit polyclonal anti-CASP8AP2 (Abcam, UK), rabbit polyclonal anti-β-actin (MBL, Japan), goat polyclonal anti-Lamin B (Santa Cruz, USA), Dylight 800 AffiniPure goat anti-rabbit IgG and anti-mouse IgG(H+L, EarthOx, USA).

### Statistics

Independent-samples T test was used to compare cell numbers in 697 cell with or without over-expression of *E2F3a* at different time points. Nonlinear regression curve fit was used to draw the curve and independent-samples T test was used to compare the regression coefficients of the two curves after VCR or DNR treatments in 697 and REH cells with or without over-expression of *E2F3a*, and in 697 cell over-expressing *E2F3a* with or without knock-down of *CASP8AP2*. Relative fluorescence intensities among the groups transfected with different plasmids were compared using one-way Analysis of Variance (ANOVA) or Fisher’s Least Significant Difference (LSD) test. It was regarded as statistically significant when *P *< 0.05. The statistical analyses were carried out using SPSS16.0 software package. The fitting curves of inhibitory effects of chemotherapeutic drugs on cell proliferation were plotted by GraphPad Prism 5, and half maximal inhibitory concentration (IC50) was also calculated by the software.

## Results

### E2F3a over-expression led to an increased fraction of leukemic cells in S and G2/M phases and accelerated proliferation

**Fig. 2:**
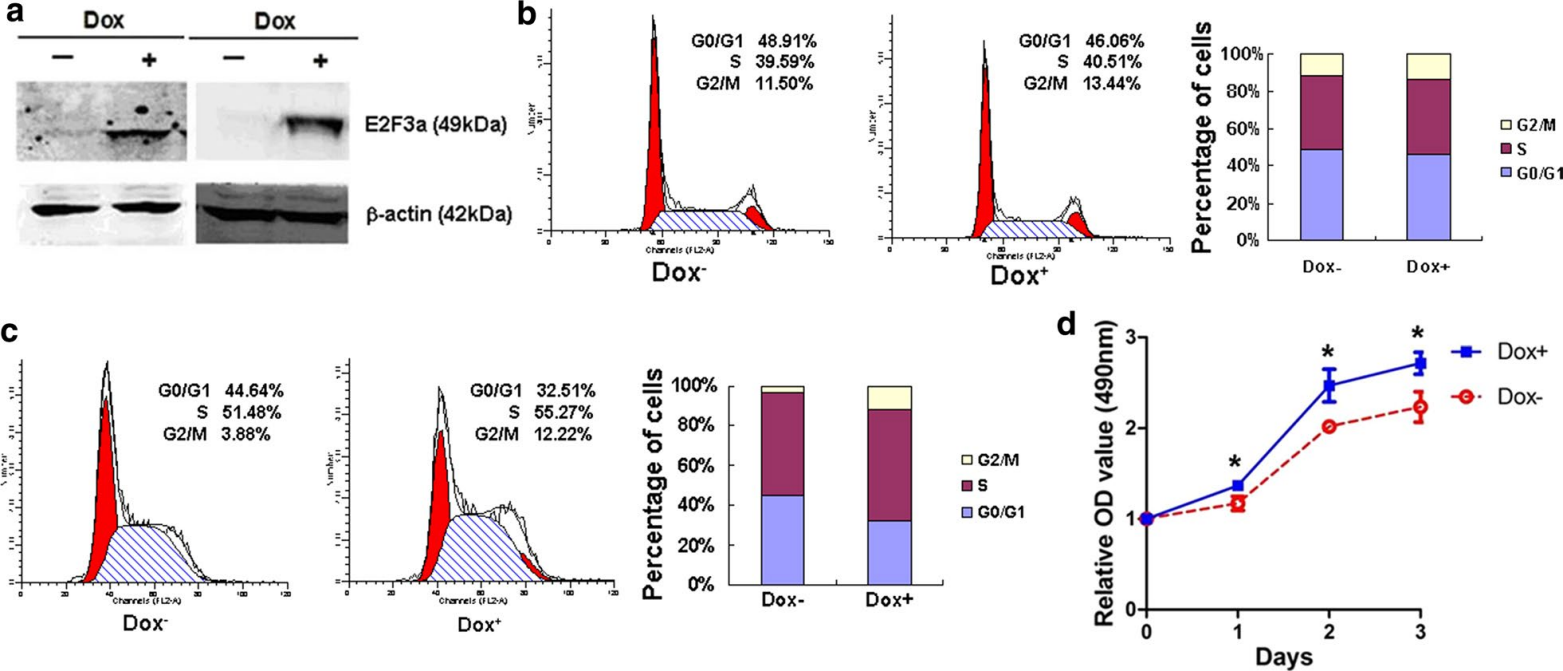
Effects of over-expression of *E2F3a* on the distribution of cell cycle phases and proliferation of 697. **a** *E2F3a* was significantly up-regulated after 24 h induction of Dox (0.5 μg/ml) in 697 (left) and REH (right) cells. **b** In 697 cells, Dox had no effect on the cell cycle phase fraction. **c** In 697 cells, over-expression of *E2F3a* resulted in decrease or increase in the fraction of cells in G0/G1, or S and G2/M phases respectively. **d** Over-expression of *E2F3a* led to accelerated proliferation. The standard errors of the means are shown (n = 3 experiments for each time point); independent-samples T test, *P < 0.05

*E2F3a* was significantly up-regulated by 24 h induction of Dox (0.5 μg/ml) in 697 stably infected with Tet-on lentivirus (Fig. 2a). Flow cytometric determination showed no effect of Dox on cell cycle phase fraction (*P *> 0.05, Fig. 2b). It was noteworthy that there was a significant decrease in the fraction of cells in G0/G1 phases upon induction of E2F3a expression by Dox (44.64% ± 2.18% vs. 32.51% ± 1.34%, *P *= 0.001). By contrast, obvious increases in S and G2/M phases cells were observed (51.48% ± 2.21% vs. 55.27% ± 0.34%, *P *= 0.043; 3.88% ± 2.43% vs. 12.22% ± 1.02%, *P *= 0.005, Fig. 2c). MTS assay also showed that after Dox induction and 1–3 days culture, the proliferation of infected cells was significantly accelerated since day 1 (*P* value was 0.007, 0.003, 0.004 for day 1–3 respectively, Fig. 2d).

### Over-expression of E2F3a enhanced sensitivity of leukemic cells to chemotherapeutic drugs vincristine and daunorubicin

**Fig. 3:**
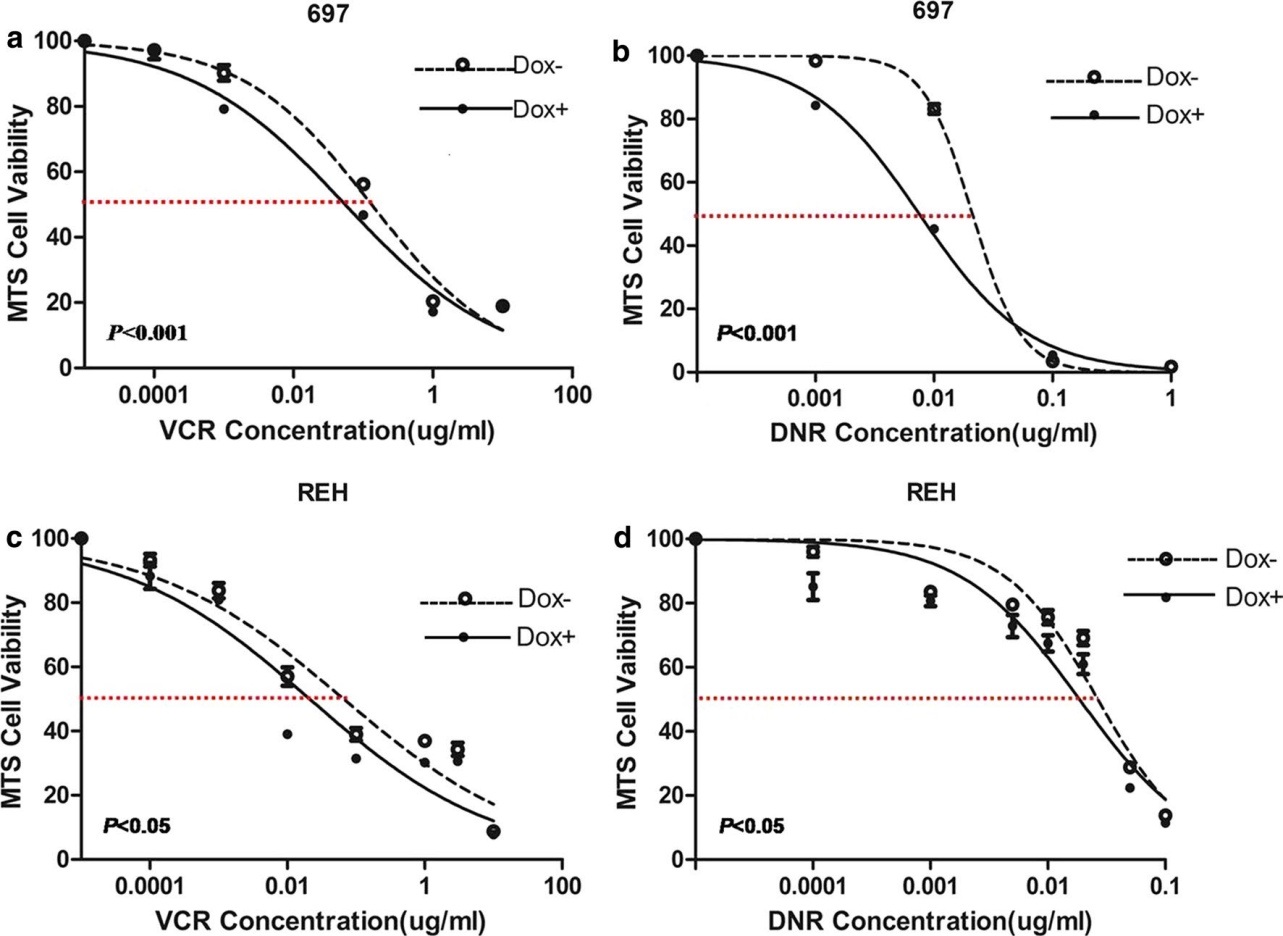
Over-expression of *E2F3a* enhanced sensitivity of 697 and REH to chemotherapeutic drugs. **a, b** Enhanced sensitivity of 697 to VCR and DNR induced by over-expression of *E2F3a* (Dox − vs. Dox + at each drug concentration, independent-samples T test was used to compare the regression coefficients of the two curves, *P *< 0.001). **c, d** Enhanced sensitivity of REH to VCR and DNR induced by over-expression of *E2F3a* (Dox − vs. Dox + at each drug concentration, independent-samples T test was used to compare the regression coefficients of the two curves, *P *< 0.05). The standard errors of the means are shown (n = 3 experiments for each drug concentration)

Vincristine (VCR) and daunorubicin (DNR) were two main chemotherapeutic drugs in induction therapy of ALL. Different concentrations of VCR and DNR were used to treat leukemic cells for 24 h. In 697 and REH cells, over-expression of *E2F3a* (Fig. 2a) could obviously decrease cell viability; half maximal inhibitory concentrations (IC50) were also reduced (Table 2, Fig. 3a–d). These suggested the role of E2F3a in enhancing sensitivity of leukemic cells to chemotherapeutic drugs.

**Table 2:** IC50 after treatment with VCR and DNR in leukemic cells over-expressing *E2F3a*

Cell lines	Drugs	*E2F3a* over-expression	IC50 (μg/ml)	*P*
697	VCR	Dox −	0.1313	< 0.001
		Dox +	0.0548	
	DNR	Dox −	0. 0211	< 0.001
		Dox +	0.0075	
REH	VCR	Dox −	0.0658	< 0.05
		Dox +	0.0211	
	DNR	Dox −	0.0266	< 0.05
		Dox +	0.0108

### E2F3a could activate *CASP8AP2* transcription

**Fig. 4:**
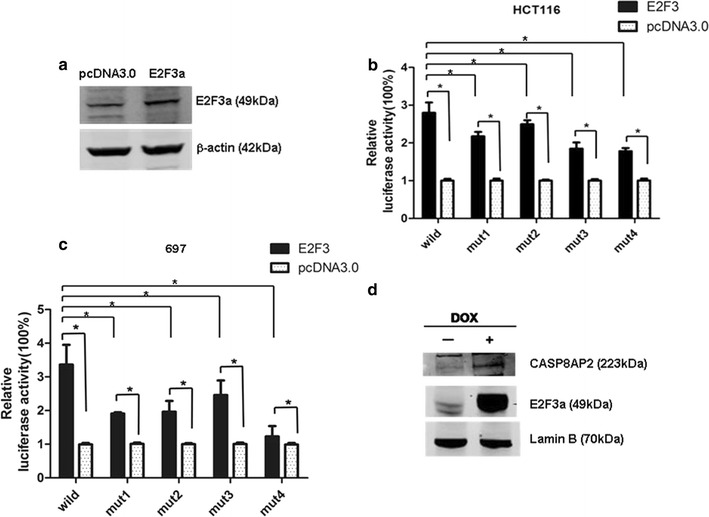
E2F3a over-expression enhanced the transcriptional activity of *CASP8AP2.* **a** E2F3a was up-regulated 24 h after transfection of pcDNA3-E2F3a in HCT116 cell. **b, c** In HCT116 and 697, relative luciferase activity of CASP8AP2-wild was higher than that of the 4 types of mutant promotor reporters when co-transfected with pcDNA3-E2F3a. The standard errors of the means are shown (n = 3 experiments for each mutant); independent-samples T test, *P < 0.05. **d** E2F3a over-expression resulted in up-regulation of CASP8AP2 in 697 cell

The leukemic cell line 697 was treated with Dox for over-expression of E2F3a, then transfected using electroporation method with plasmid CASP8AP2-wild. For HCT116, pcDNA3-E2F3a and CASP8AP2-wild were co-transfected using Lipofectin method. When *E2F3a* was induced to over-express (Figs. 2a, 4a), the relative luciferase activity of the wild type of CASP8AP2 promotor was increased significantly (*P *< 0.05, Fig. 4b, c). When pcDNA3-E2F3a was co-transfected into HCT116 or 697 with CASP8AP2-mutant 1–4 respectively, the luciferase activities of all the mutated types of promotors were obviously enhanced, however, all were lower than the wild type (*P* < 0.05 for all, Fig. 4b, c). These results indicated that E2F3a was able to enhance the transcriptional activity of *CASP8AP2*; the three E2F binding sites at − 614, − 169, and − 131 were all involved in the activation role of E2F3a. Accordingly, over-expression of E2F3a resulted in significant up-regulation of CASP8AP2 in 697 cell (Fig. 4d).

### E2F3a could bind *CASP8AP2* promoter directly

**Fig. 5:**
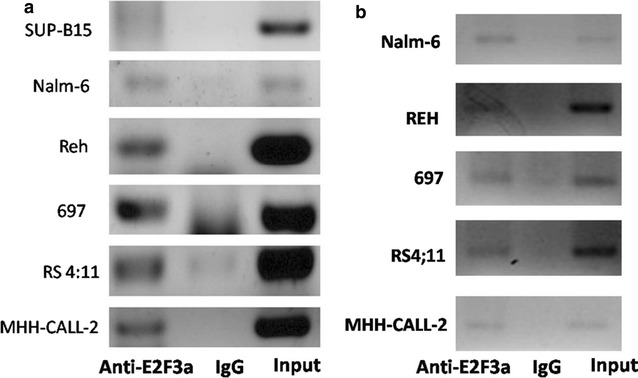
E2F3a could bind the two fragments of − 206 to − 69 (**a**) and − 677 to − 507 (**b**) in *CASP8AP2* promotor

ChIP assay was used to determine whether endogenous E2F3a was capable of binding the two fragments (− 206 to − 69 and − 677 to − 507) of *CASP8AP2* promotor directly in six leukemic cell lines, including MHH-CALL-2, REH, RS4;11, SUP-B15, Nalm-6 and 697. In all the cell lines, there were clear specific bands in the samples of input and anti-E2F3a, whereas no or weak bands were noticed in the control samples (Fig. 5). Sequencing and alignments indicated that the amplification products matched the sequences of *CASP8AP2* promoter completely. Together, these results showed that E2F3a was able to directly bind the two promoter regions of *CASP8AP2*.

### Down-regulation of *CASP8AP2* reversed the advancing effect of E2F3a on chemotherapeutic sensitivity of 697 cell

As described above, E2F3a could enhance the sensitivity of 697 and REH cells to chemotherapeutic drugs. It was noticed that high expressions of both *E2F3a* and *CASP8AP2* were associated with better treatment response and prognosis; moreover, E2F3a could bind *CASP8AP2* promotor and activate its transcription. Thus, the role of CASP8AP2 played in the effects of E2F3a on chemotherapeutic sensitivity was investigated further.

**Fig. 6:**
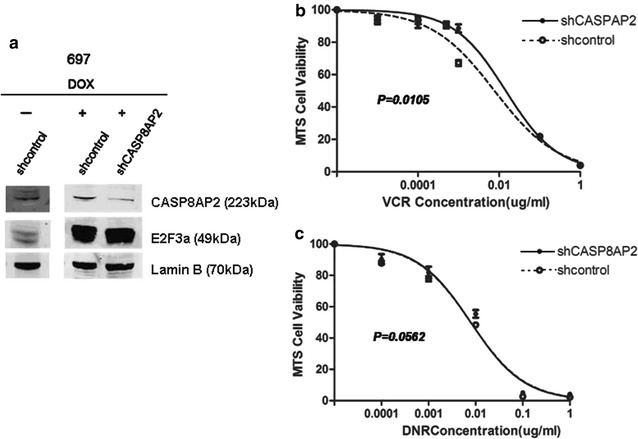
Knockdown of CASP8AP2 reversed the effect of E2F3a on chemotherapeutic sensitivity of 697. **a** CASP8AP2 knockdown with shCASP8AP2 in 697 over-expressing E2F3a. **b** IC50 of VCR was increased in 697 over-expressing E2F3a after CASP8AP2 knockdown (*P *= 0.0105). **c** IC50 of DNR tended to increase in 697 over-expressing E2F3a after CASP8AP2 knockdown (*P *= 0.0562). Independent-samples T test was used to compare the regression coefficients of the two curves. The standard errors of the means are shown (n = 3 experiments for each drug concentration)

shCASP8AP2 led to down-regulation of CASP8AP2 in 697 stably infected with Tet-on E2F3a lentivirus (Fig. 6a). When these cells were treated by different concentrations of VCR, the cell viability and IC50 were significantly increased (Fig. 6b, shControl vs. shCASP8AP2, 0.0076 vs. 0.0145, *P *= 0.0105). Increase tendency was also found after treatment with DNR (Fig. 6c, shControl vs. shCASP8AP2, 0.0173 vs. 0.0188, *P *= 0.056). Taken together, the advancing effect of E2F3a on chemotherapeutic sensitivity of ALL cells was implemented by regulating *CASP8AP2* expression to a considerable degree, and could be reversed by down-regulating *CASP8AP2*.

## Discussion

The expression level of CASP8AP2 was an important independent prognostic factor in childhood ALL. The patients with low expression of CASP8AP2 at diagnosis were at greater risk of relapse [11]. However, it was unclear about the mechanisms of low expression of *CASP8AP2* in childhood ALL. Our previous study has shown that in childhood ALL, hypermethylation of two CpG sites located at − 1189 and − 1176 in *CASP8AP2* promotor was correlated to mRNA low expression, MRD positivity before consolidation therapy (day 78th) and poor prognosis [21]. It was worthy to note that the methylation of these two CpG sites was not the key factor to affect gene expression. Since the methylation of core promotor of *CASP8AP2* decreased gene expression and induced resistance to chemotherapeutic drugs [22, 23], it was deduced that the specific transcription factors capable of binding core promotor and inducing transcription played the pivotal role in regulating *CASP8AP2* expression. Thus, the key to clarify the mechanism of low expression of *CASP8AP2* in childhood ALL was to identify the transcription factors capable of binding core promotor of *CASP8AP2*.

There were three binding sites of E2F located at − 614, − 169, and − 131 in *CASP8AP2* promotor. The studies of gene expression profiling of St. Jude Children’s Research Hospital and us have indicated that only E2F3 expressed, whereas the expression of other members including E2F1, E2F2, and E2F 4–6 could not be detected in most samples [15, 16]. Therefore, E2F3a was probably an important factor to regulate *CASP8AP2* expression in childhood ALL. In present study, E2F3a over-expression could activate *CASP8AP2* transcription; mutations of the three E2F binding sites at − 614, − 169, and − 131 made transcription activity decreased significantly. Meanwhile, the binding of E2F3a with the two fragments of CASP8AP2 promotor, − 206 to − 69 and − 677 to − 507, was identified by ChIP (Fig. 5). Thus, this study uncovered the fact that E2F3a was able to bind *CASP8AP2* promotor and activate its transcription. It should be noted that as dual luciferase assay showed, relative luciferase activity was still higher in the reporter with three mutations at − 614, − 169, and − 131 than in the control. This was probably due to the existence of other binding sites of some transcription factors, such as c-Myb, c-Myc, and AP-1, which had the potential activating function on the promotor. Thus, further studies were needed to clarify the mechanism of low expression of *CASP8AP2* in childhood ALL.

E2F3a was the key factor of controlling DNA replication and transition of G1 to S phase [19]. In the other hand, CASP8AP2 was essential to progression of S phase, transcription and 3′end processing of replication dependent histone mRNA [5, 7]. In this scenario, the positive regulation of E2F3a on *CASP8AP2* transcription could coordinate genomic DNA replication and histone biosynthesis, with the result of chromatin integrity and stability. The similar results in colorectal cancer cells and leukemic cells suggested that this regulation probably existed in many types of tissues and cells, and was essential to cell proliferation.

Abnormal high expression of *E2F3a* has been found in many types of solid tumors [13, 14, 19], often associated with poor prognosis [24, 25]. However, our previous study has shown that *E2F3a* was generally low-expressed and associated with leukemic relapse or induction failure in childhood ALL [20]. Accordingly, in present study, we showed that *E2F3a* over-expression made leukemic cells proliferate more rapidly, and more sensitive to VCR and DNR. It has been long recognized that slow-proliferating leukemic cells were resistant to common chemotherapeutic drugs including VCR, DNR, cytarabine, methotrexate, and thioguanines [26]. As E2F3a plays an important role in G1/S transition and cell proliferation [19], its down-regulation probably led to slow proliferation and chemotherapeutic resistance of leukemic cells in childhood ALL. Many groups have reported similar results. Flotho et al. have reported the association of the low expression of some genes essential for cell cycle progress with the high level of MRD and relapse in childhood ALL, such as *CCNB2*, *CDC2*, *CKS1B*, and interestingly, *CASP8AP2* [8, 9].

It was noteworthy that although E2F3a overexpression reduced IC50 of leukemic cells, there was no significant difference in the viability of leukemic cells at high concentrations of chemotherapeutic drugs. It meant that the effect of E2F3a expression level on chemoresistance was related to drug concentration. High concentration of drugs or combined chemotherapy would play a bigger role in eliminating leukemic cells.

Although our results were contrary to that of studies in solid tumors; the reason behind this contradiction was unclear. Based on the results of present study, down-regulation of *CASP8AP2* which was also associated with leukemic relapse in childhood ALL [11] may be one of the main reasons of chemotherapeutic resistance of low-expression of *E2F3a*.

To test this hypothesis, *CASP8AP2* was knocked down in 697 with over-expression of *E2F3a* and sensitivity to VCR and DNR was determined. As expected, IC50 of VCR and DNR treatment significantly increased or tended to increase respectively. Therefore the advancing effects of E2F3a on sensitivity to chemotherapeutic drugs were implemented via up-regulating *CASP8AP2* to a great extent. On the other hand, as VCR and DNR were cell cycle specific or non-specific drugs respectively, these results also suggested that the effects of *CASP8AP2* on sensitivity to drugs was mainly implemented via influencing cell cycle progression, whereas E2F3a may make effects on chemotherapeutic sensitivity via other ways to be investigated.

Many mechanisms and factors were involved in resistance to chemotherapeutic drugs of tumor cells, such as DNA damage and repair, cell apoptosis, metabolism and absorption of drugs, and abnormal cell cycle [27]. Except the effects on cell cycle progression revealed in the present study, there may be other ways by which E2F3a and CASP8AP2 affected chemotherapeutic sensitivity in childhood ALL. In these processes, interactions of E2F3a and CASP8AP2 with other proteins needed to be clarified. In addition, development of novel small molecules and techniques for target therapy against low expression of E2F3a and *CASP8AP2* was an important direction for further study.

## Conclusions

Our data suggest that E2F3a increased leukemic cells in S and G2/M phases, accelerated proliferation and enhanced sensitivity to chemotherapeutic drugs. Furthermore, E2F3a could bind *CASP8AP2* promoter directly and activate *CASP8AP2* transcription; CASP8AP2 down-regulation reversed the advancing effect of E2F3a on chemosensitivity.

## Abbreviations

*CASP8AP2*: caspase 8 associated protein 2
ALL: acute lymphoblastic leukemia
ChIP: chromatin immunoprecipitation
EFS: event free survival
MRD: minimal residual disease
Tet-on shRNA: tetracycline-inducible short hairpin RNA
FBS: fetal bovine serum
IMDM: Iscove’s modified Dulbecco’s media
FL: firefly luciferase
RL: renilla luciferase
Dox: doxycycline
PI: propidium iodide
ANOVA: analysis of Variance
LSD: Fisher’s Least Significant Difference
VCR: vincristine
DNR: daunorubicin
IC50: half maximal inhibitory concentrations
